# The Efficacy of Surgical Techniques for the Management of Confirmed Vertical Root Fractures: A Systematic Review

**DOI:** 10.1111/aej.12923

**Published:** 2025-02-01

**Authors:** Stefano Corbella, Silvio Taschieri, Igor Tsesis, Tomer Goldberger, Luca Francetti, Eyal Rosen

**Affiliations:** ^1^ Department of Biomedical, Surgical and Dental Sciences Università Degli Studi di Milano Milan Italy; ^2^ IRCCS Ospedale Galeazzi Sant'Ambrogio Milan Italy; ^3^ Department of Endodontics The Maurice and Gabriela Goldschleger School of Dental Medicine, Faculty of Medicine, Tel Aviv University Tel Aviv Israel

**Keywords:** dental bonding, oral surgery, systematic review, tooth fractures, tooth root fractures

## Abstract

The aim of the present systematic review was to evaluate the efficacy of surgical techniques for the preservation of a tooth with VRF. We included case series presenting techniques for the treatment of VRFs. Both electronic sources (MEDLINE, EMBASE, Cochrane Central) and reference lists/table of contents of pertinent journals were screened. Eight articles (six studies) were included. Four of the studies bonded the fractured fragments with 4‐META/MMA‐TBB resin, one adopted MTA, and in one study the authors used resin‐ionomer cement. There was significant heterogeneity in the results regarding teeth survival rate and it was not possible to perform a quantitative synthesis. Scientific evidence concerning the treatment of vertically fractured roots is sparse and of low quality. Consequently, it is not possible to draw conclusions regarding the efficacy of the reported techniques. More studies with higher scientific standards may add validity to the techniques described here.

**Trial Registration:** CRD42024524356 in PROSPERO

## 1 Introduction

Vertical root fractures (VRFs) can be defined as axial fractures involving all the structures of the tooth root, namely the cementum, the dentin and the root canal system [1]. The lesion may originate in either the apical or cervical regions of the root, depending on the causal factor, and may be incomplete (only one side of the root), or complete (both sides) [1]. VRFs usually occur in the bucco‐lingual direction but they may also be oriented in the mesio‐distal direction, depending on tooth root characteristics, and anatomy [2]. The damage may evolve into a split tooth, with complete separation of the tooth structure [3].

The presence of post restoration, the amount of residual dentin, and other factors related to tooth anatomy have all been described as possible predisposing risk factors for VRFs [1, 4]. However, a recent systematic review of the literature concluded that there is currently insufficient evidence for the influence of local and systemic factors (gender, tooth location and type, tooth restoration) on the development of the condition [2]. Although VRFs occur primarily in endodontically treated teeth, they have also been described, albeit rarely, in vital teeth [5, 6]. There is some evidence that maxillary premolars and molars are more prone to develop VRFs than other teeth, probably due to the anatomical characteristics of the roots [7, 8].

The diagnosis of VRF can be challenging, especially at the beginning, because of the variability in presentation. Rivera and Walton reported that the initial signs and symptoms of VRFs may be none to slight [3]. However, once bacteria penetrate the fracture, signs of apical periodontitis (AP), tenderness to percussion, swelling, bleeding and pain on biting may appear [1, 6, 9]. The most characteristic clinical sign of VRF is a deep, narrow and isolated periodontal probing depth, not due to other conditions (e.g., periodontal disease), which follows the fracture itself [6, 9]. In some cases, it is possible to identify an associated sinus tract located in mid‐root level [10].

One pivotal study by Tamse and coworkers published in 1999 [11] described the radiographic characteristics of teeth with VRF on periapical bidimensional radiographs and concluded that the most common features are ‘halo’ radiolucency, and an apico‐lateral (J‐shaped) radiographic lesion, often involving the area of furcation. Although CBCT could be useful for diagnosis or preclinical planning of endodontic treatment when periapical bidimensional radiographic data are inconclusive, [12], the observed accuracy does not support the routine use of this tool for the diagnosis of VRFs [12, 13, 14]. When clinical and radiographic signs are not sufficient for diagnosis, the confirmation of the presence of a fracture may be achieved through direct visualisation after surgical flap elevation [15] or after extracting the affected root.

A VRF may rapidly evolve to produce a complete fracture, which splits the root into two or more fragments and causes severe peri‐radicular infection [1]. This may then require extraction and, eventual replacement of the lost teeth [16]. Although in multi‐rooted tooth partial or complete amputation of the affected root may enable preservation of the tooth [17], single‐rooted teeth with VRF are often considered a ‘hopeless’ clinical situation. Several studies, mostly case reports that describe the treatment of such fractured roots with both intraoral and extraoral surgical techniques, have very variable starting conditions and results [4, 18, 19]. We did not find a systematic meta‐analysis of surgical techniques and methods for the management of roots with VRF in the literature. Therefore, the present systematic literature review was designed to fill this gap by evaluating the efficacy of different surgical techniques and methods for the clinical management and preservation of roots with VRFs.

## 2 Methods

The protocol of the study was registered in PROSPERO (Registration number CRD42024524356) and the present manuscript was written following the PRISMA (preferred reporting items for systematic reviews and meta‐analyses) statement [20]. The protocol followed the instructions provided by the Cochrane Handbook for Systematic Review of Interventions—Second Edition [21].

The main objective of the present systematic review was to answer the following focused question: *what is the efficacy of different surgical techniques for the preservation of the fractured root in patients with confirmed vertical root fracture. The outcome of tooth survival was based on the analysis of relevant randomised controlled clinical trials, nonrandomised controlled trials and prospective and retrospective case series*.

### 2.1 Eligibility Criteria

Based on the PICOS framework, the criteria for considering the studies were:

- *Population (P)*: ≥ 18 years old, presenting one tooth with a diagnosis of vertical root fracture as defined by the criteria of Patel and coworkers [1]. This should have been diagnosed preliminarily by clinical and/or radiographic examination and could be confirmed during the intervention.
- *Intervention (I)*: any surgical technique designed to preserve the fractured root.
- *Control (C)*: other techniques or the same technique employed as intervention but with modifications in the protocol or in the materials adopted.
- *Outcomes (O)*: the primary outcome is tooth survival. The secondary outcomes are the occurrence of adverse events or complications and, if present, patient‐reported outcomes (PROMS).
- *Studies (S)*: randomised controlled clinical trials, nonrandomised controlled clinical trials and prospective or retrospective cases series.

Case reports and studies reporting less than five treated patients were excluded.

### 2.2 Sources and Search Strategy

The following electronic search engines were searched for articles pertinent to the study: MEDLINE through OVID interface, EMBASE, Cochrane Central. The search strategy is presented in detail in Appendix A. The grey literature was searched by interrogating Greylit and OpenGrey. The authors also screened trials registers (ClinicalTrials.gov and EU Clinical Trials Register).

A manual search of all potentially relevant articles was performed by screening the reference lists of all included manuscripts and all issues (published since 2000) of the *International Endodontic Journal*, *Journal of Endodontics*, *Australian Endodontic Journal*, *Journal of Dentistry* and *Journal of Dental Research*. We excluded conference papers and abstracts, but we posed no language restrictions.

The last electronic search was performed on 5th January 2024.

### 2.3 Selection Process

Two authors (ST, SC) independently screened titles and abstracts for the inclusion criteria. The full text of all preselected papers was then retrieved and again screened carefully in order to confirm inclusion or exclusion. In case of disagreement, the dispute was resolved by consulting a third reviewer (ER). The level of concordance in each step was measured by Cohen's kappa and recorded.

### 2.4 Data Extraction

Two authors (ST, SC) extracted the following items, when available, from the papers: authors' names, year of publication, country, study type, characteristics of the sample (age distribution, sex distribution, ethnicity, educational status, smoking status), the setting, number of patients/teeth treated, description of the techniques, VRF description, clinical results, patient‐reported outcomes, adverse events.

In case of missing information, it was planned to contact the authors (maximum of twice) by email in order to obtain data.

### 2.5 Risk of Bias Evaluation and Quality of Evidence Assessment

The methodological quality of case series studies was evaluated by using the JBI Critical Appraisal Tool for case series [22].

### 2.6 Data Synthesis and Analysis

Due to the characteristics of the studies included in the review, a comparative meta‐analysis was not feasible and for this reason, a quantitative integration of the results was not performed. Instead, the data from single studies were considered.

## 3 Results

**FIGURE 1:**
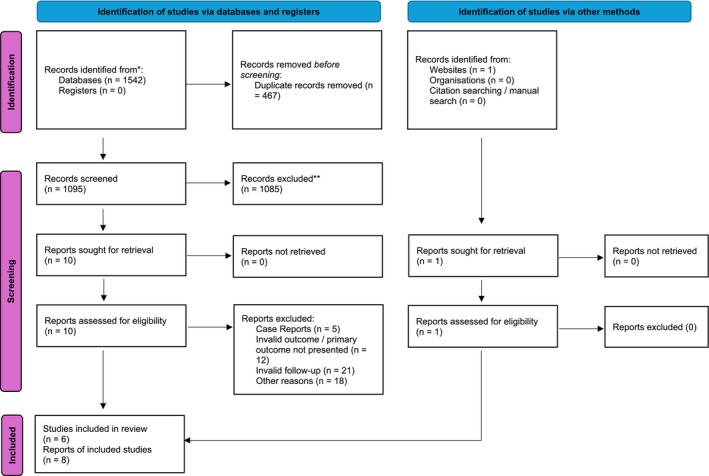
PRISMA diagram of article selection flow [20].

The literature search retrieved a total of eight articles (six studies) that were included and considered in the present review. The summary of article selection process is summarised in Figure 1. The general characteristics of the included papers are summarised in Table 1. All the included studies are case series, and two present comparative data [4, 23]. For the purposes of the present review, in the study by Sugaya [4], we considered only the group treated by a surgical approach (extraction, repair and replantation). Two studies were performed in Japan [4, 23], two in the United States [24, 25], one in Italy [26, 27] and one in Taiwan [18]. Two studies were performed in a university setting, and two in a private clinic setting.

**TABLE 1:** General characteristics of the included studies.

ID study	ID article	Authors	Year	Study type	Country/Population	Setting	No patients/No teeth	Techniques	VRF type	Results
1	1	Hayashi M et al.	2002	CS	Japan	University Hospital	20 patients/20 teeth (part of the same cohort of Hayashi et al. [28])	Technique 1: (HAIRLINE CRACKS) local anaesthesia, tooth extraction, extracted teeth kept moist with sterile saline, socket debridement, crack sealed extraorally with 4‐META/MMA‐TBB dentin‐bonded resin after shallow preparation with diamond points, teeth replanted, restored with metallic post and crown Technique 2: (INCOMPLETE AND COMPLETE FRACTURE) local anaesthesia, fragments extraction, fragments adhered extraorally with 4‐META/MMA‐TBB dentin‐bonded resin after cleaning with ultrasonic, kept in gauze for 5 mins after adhesion, then replanted, and restored with a metallic post and crown	See Hayashi et al. [28]	See Hayashi et al. [28]
1	2	Hayashi M et al.	2004	CS	Japan	University Hospital	26 patients/26 teeth	See Hayashi et al. [23]	8 hairline cracks, 9 complete fractures, 2 separated, 7 incomplete fractures	8 teeth extracted (6 premolars, 2 M; 1 for technique 1, 7 for technique; 6 with fracture located in apical region, 2 with fracture located in middle region)
									8 incisors (3 hairline, 2 complete, 1 separate, 2 incomplete); 14 premolars (3 hairline, 6 complete, 1 separate, 4 incomplete); 4 M (2 hairline, 1 complete, 1 incomplete)	6 successes (3 incisors, 3 premolars; 2 for technique 1, 4 for technique 2; 3 with fracture located in apical region, 3 with fracture located in middle region)
									No information about symptomatic/asymptomatic	12 functional, with periapical lesion (5 incisors, 5 premolars, 2 M; 4 for technique 1, 8 for technique 2; 3 with fracture located in apical region, 7 with fracture located in middle region, 2 with fracture located in cervical region)
										Mean follow‐up 34.9 months (47.9 months incisors, 29.5 months premolars, 36.5 M; 50.6 months technique 1, 27.9 months technique 2)
										Mean time 34.8 mins (37.5 mins incisors, 34.4 mins premolars, 40 mins molars; 35.6 mins technique 1, 34.4 mins technique 2)
2	3	Selden H	1996	CS	United States	University Hospital	6 patients/6 teeth	Full‐thickness flap, removal of granulation tissues, pressure with gauze sponges, visualisation with microscopy, excavation of the VRF with an ultrasonic tip to a depth of 2–3 mm and a width of 1 mm, defect bonded with 4‐META, fracture packed with silver glass‐ionomer cement, wetted hydroxylapatite, non‐resorbable membrane e‐PTFE, sutured with Gore‐tex sutures	All incomplete vertical root fractures	Five roots failed after 2–11 months from treatment; one survived at the 12‐month follow‐up but split
								3 roots treated as above, 2 with small differences in the material used for repair, one treated as above but without GBR	No information about symptomatic/asymptomatic	
3	4	Okaguchi M et al.	2019	CS	Taiwan	Private dental clinic	6 patients/6 teeth	Intentional replantation and root fragment bonding with 4‐META/MMA‐TBB resin. The technique is the same used in Hayashi et al. [23]	Complete vertical root fractures in bucco‐lingual direction; Five teeth with periradicular radiolucency	Follow‐up 33 to 74 months (mean 49.5 + − 15 months)
									No information about symptomatic/asymptomatic	Radiographic healing: 4 complete healing, 1 nearly complete healing, 1 not assessed. Clinical outcome: 6 successful
4	5	Taschieri S et al.	2009	CS	Italy	Private dental clinic	9 patients/9 teeth	Local anaesthesia, trapezoidal PBI flap, visualisation with surgical loupes, removal of granulation tissue, detection of the fracture by using microscope and methylene blue dye, the fracture was instrumented with zirconium nitride retro‐tips on ultrasonic device, the groove was treated with 17% EDTA, sealed and filled with MTA, bone buccal defect filled with calcium sulphate, flap sutured 5/0 sutures	Incomplete VRF. 4 central incisors, 2 lateral incisors, 2 canines, 1 premolar, all maxillary Symptomatic	See Taschieri et al. [26]
4	6	Taschieri et al.	2010	CS	Italy	Private dental clinic	10 patients/10 teeth	See Taschieri et al. [27]	Incomplete VRF. 4 central incisors, 3 lateral incisors, 2 canines, 1 premolar, all maxillary Symptomatic	All successful after 1 year. After 33 months 5/7 teeth resulted successful while 2/7 were extracted (lateral incisors)
5	7	Sugaya et al.	2001	CS	Japan	NS	12 patients/12 teeth*	Root fragments extracted, cleaned with burs, etched with citric acid, washed, dried, bonded with 4‐META/MMA‐TBB resin, fragments pressed together, replantation after debridement of the socket, splinted to the proximal teeth	7 complete fractures, 5 incomplete fractures; 2 incisors, 2 canines, 3 premolars, 5 M; 5 full cast crowns, 5 fixed bridges, 2 coping No information about symptomatic/asymptomatic	Group B: 9 good prognosis (mean follow‐up > 22 months), 3 extracted due to deep pocket/mobility and refracture
6	8	Anderegg et al.	2018	CS	United States	NS	5 patients/5 teeth	Full‐thickness flap, periodontal debridement, VRF repaired with an inverted cone bur, defect filled with a resin‐ionomer, flap sutured with 4/0 sutures	1 central incisor, 2 lateral incisors, 2 canines No information about symptomatic/asymptomatic	All failed within 6 months from the intervention

Abbreviation: NS, not specified.

* Only group B was considered, following the inclusion criteria.

The fractured fragments in four studies were bonded with 4‐META/MMA‐TBB resin, compared to MTA in one study, and resin‐ionomer cement in the last study. Four studies described a technique for VRF repair without extraction [24, 25, 26, 27].

The outcomes of the quality assessment are presented in Table 2. The items that most negatively affected the quality appraisal were the lack of complete demographic information about the patients and the depth of the planned statistical analysis, which was also limited by the low number of subjects included. Moreover, all studies but one [26, 27] did not report the presence/absence of symptoms.

**TABLE 2:** Quality appraisal.

	Clear inclusion criteria	Standard reliable method for measuring the condition	Valid methods for identification of the condition	Consecutive inclusion of participants	Complete inclusion of participants	Clear reporting of demographics	Clear reporting of clinical information	Outcomes or follow‐up results clearly reported	Clear reporting of the presenting sites/clinics demographics	Statistical analysis appropriate
Hayashi M et al.	Yes	Yes	Yes	Yes	Unclear	No	Yes	Yes	Not applicable	Not applicable
Selden H	Yes	Not applicable	Yes	Unclear	Unclear	No	Yes	Yes	Not applicable	Not applicable
Okaguchi M et al.	Yes	Yes	Yes	Unclear	Unclear	No	Yes	Yes	Not applicable	Not applicable
Taschieri S et al.	Yes	Unclear	Yes	Unclear	Unclear	No	No	Yes	Not applicable	Not applicable
Sugaya et al.	Yes	Yes	Yes	Unclear	Unclear	No	Yes	Yes	Not applicable	Yes
Anderegg et al.	Yes	Unclear	Yes	Unclear	Unclear	No	No	Yes	Not applicable	Not applicable

The characteristics of the studies did not allow a quantitative analysis of the results, so a qualitative description of the outcomes of the included study is presented.

The study by Hayashi and coworkers [23, 28] compared management of VRF by extracting or not extracting the fragments and managing the fracture outside, or inside the mouth, respectively. Eight of 26 initially treated teeth (7 of the teeth bonded after extraction and then replanted, and 1 of the teeth bonded without extraction) failed after a mean follow‐up of 34.9 months. Their results indicated significantly lower success for posterior teeth and for teeth with longitudinal fractures extending more than 2/3 from the cervical portion.

The study by Sugaya and coworkers, which compared an extraoral technique for bonding fractured teeth fragments with 4‐META/MMA‐TBB resin, reported that 9 of the 12 treated teeth were still functional after 22 months, with a good prognosis, and without any evidence for a difference between the two techniques [4]. Furthermore, by 6 months after treatment the mean probing depth was reduced from 7.4 mm at pre‐treatment to 4.6 mm, the bleeding scores lowered from 91% to 8.3% and the radiographic bone level increased from 18.8% to 29.2%. There were no ankylosed teeth nor was any root resorption detected. They concluded that the treatment of vertical root fracture using extra‐oral resin bonding and replantation has good prognostic possibilities.

The study by Okaguchi and coworkers, who employed an extraoral protocol of bonding and subsequent replantation of fractured teeth, reported a successful outcome in all the teeth of six patients after 33–74 (mean 50 ± 15) months, with four showing complete healing [18]. The authors concluded that intentional replantation combined with root fragment resin bonding is a successful treatment modality that can be used to preserve a complete VRF tooth.

In the study published in 1996 Selden performed the conservative treatment of six teeth with incomplete VRF using silver glass ionomer cement with bone graft, where all cases failed in the long term [24].

Two studies on the same cohort of patients evaluated a modern surgical technique for repairing incomplete VRF on maxillary anterior teeth by using mineral trioxide aggregate (MTA) to seal the fracture, followed by filling the bone defect with calcium sulfate. In their case, the treatment of five out of seven teeth was successful after 33 months, although two had required extraction [26, 27]. Notably teeth with deep probing depths (> 4 mm) and teeth with certain radiographic appearances (such as interproximal angular radiolucency on one side of the root and halo‐like periradicular radiolucency) were excluded from this study.

In another recent study, after the fractured root area was exposed using a full‐thickness mucogingival flap, followed by periodontal debridement and repair of the fracture by enlarging the fracture with an inverted cone bur and filling the defect with a resin‐ionomer, the authors concluded that ‘clinicians should be cautious in deciding to attempt repair of a VRF by using resin‐ionomer cements’ [25]. If such a technique is utilised, they advised frequent recall to detect potential patterns of continued bone loss, progression of chronic infection at the fracture site and possible case failure.

## 4 Discussion

The results of the present systematic review of the literature do not allow us to draw any evidence‐based conclusion about the efficacy of surgical technique for maintaining roots with longitudinal fractures.

The main limitation of the studies included is that they are all observational case series, with limited sample size. Moreover, there was a significant heterogeneity, with respect to the techniques employed. However, the systematic review of the topic in the existing literature does enable a qualitative appraisal of the current knowledge.

Currently, VRFs are usually considered an absolute indication for tooth extraction and, eventually, replacement with a prosthesis [3, 16, 29]. In some cases, the presence of incomplete, apically located, VRFs may be treated through root amputation, by removing portions of the affected root; this approach may find an application in multi‐rooted teeth, in which the resection of one root may allow the retention of the tooth [17]. Another factor that may worsen the prognosis of teeth with VRFs can be the presence of symptoms, which may occur in later phases, and can be related to the presence of abscess and mobility [1].

Repair of such lesions may only be feasible for incomplete VRFs, which should be treated promptly to prevent progression. Most literature reports about treatment of VRFs are single case reports, as also noted previously by Patel [1]. These were not considered in the present systematic review because of the limitations in generalisability of the results [30]. Similarly, while descriptive case series studies may have the advantage of presenting more data about innovative treatment approaches than single case reports and may trigger further investigations, the results cannot be used to draw any conclusion about causation, and inferences may not be justified [31].

The treatment modalities reported in the included articles may be divided into two main categories: extraction and replantation of the involved root or tooth following extraoral repair of the fracture using adhesive 4‐META/MMA‐TBB resin; and repair of the fracture (with bioceramic material) using a flap elevation procedure while the tooth remains attached to the periodontium.

It should be noted that intentional tooth replantation may be considered as a treatment option in some clinical conditions when cases cannot be managed nonsurgically or surgically, due to a number of situations such as the risk of neurovascular damages, lack of accessibility, or other options [32, 33]. Although the available evidence on this clinical procedure is based on observational studies, the reported overall retention rate ranged from 73% after 11 years to 93% after 12 years, with a relatively low rate of complications [32]. Considering periodontally compromised teeth, a recent paper reported that teeth with a periodontal—endodontic lesion (like teeth presenting a VRF) maintained survival rates of 93%, 65%, and 36.9% at 1, 5 and 10 years, respectively, which are lower than for periodontally healthy teeth [34].

In the included studies, the protocols adopted were similar but not totally identically. In particular, the methods used for extraction and to treat the fracture differed significantly and this can be one of the causes of heterogeneity and substantially reduced the external validity of the results. However, we should report that the number of adverse reactions and complications that were reported was close to nil.

The second technique that was studied was the direct repair of the fracture, after gaining access to the lesion through a surgical approach. The use of MTA for repairing perforations and external resorption, obtaining access to the lesion through flap elevation, is documented in scientific literature that reported good results and biocompatibility of the material used [35]. In the included studies, one used MTA for repairing VRFs reporting acceptable clinical outcomes [27, 36], and one repaired the fracture by using glass‐ionomer cement [24, 25]. The use of glass ionomer cements was also described in surgical endodontics [37, 38]. While some authors have advocated good clinical performances of such material when used for repairing perforations or root resorptions [37], the results of the studies included in the present review do not support the use of such types of cement for repairing VRFs [24, 25].

Through the analysis of the included studies, most of them did not report the causes of failure but we can hypothesise that in most cases complete fracture with split tooth occurred in failed cases. For such reason, in order to perform any technique that does not imply the extraction and replantation of the tooth, the presence of an incomplete VRF must be diagnosed correctly. As known, CBCT may not be useful for detecting the presence of a VRF, being more accurate than periapical radiographs but unable to visualise directly the fracture in all cases [13, 39, 40]. Presurgical clinical examination through periodontal probing should be considered as the standard diagnostic tool for detecting the presence of one deep and narrow periodontal defect potentially associated to the development of VRF. When such evidence could be found only on one side of the tooth and not on the opposite one, we can hypothesise that the fracture is indeed incomplete [41].

## 5 Conclusions

In conclusion, the current evidence about surgical treatment of VRFs is not sufficient to support the use of the described techniques as standard approaches in cases presenting with a confirmed fracture of the root. Further validation of the proposed techniques from randomised comparative clinical trials will be needed, even if the follow‐up is short, but the description of the case characteristics is accurate, since the variability noted in the studies reviewed here may be the cause of the heterogeneity in the treatment results.

## Author Contributions

Stefano Corbella prepared the study protocol, performed the search, screened the articles and drafted the paper; Silvio Taschieri approved the protocol, screened the articles and contributed to drafting the paper; Igor Tsesis approved the final manuscript and contributed to data interpretation; Tomer Goldberger approved the protocol and approved the final manuscript; Luca Francetti contributed to preparing the protocol and approved the final manuscript; Eyal Rosen prepared the protocol, contributed to data interpretation and contributed to drafting the paper.

## Conflicts of Interest

The authors declare no conflicts of interest.

## Appendix A Search Strategy

MEDLINE via OVID (4th January 2024): 657

**Table:**

	MeSH terms	Free‐text search
Population	(‘Tooth’/Exp AND ‘fracture’/Exp)	(Vertical OR Longitudinal) AND ((root OR tooth) ADJ2 (fracture* OR injur*)) OR (craze ADJ2 line*) OR (crazy ADJ2 line*)
Intervention	Exp Treatment OR Oral surgery	Treatment* OR repair* OR management OR therap* OR bonding

EMBASE (4th January 2024): 885

**Table:**

	EMTREE terms	Free‐text search
Population	‘Tooth fracture’/Exp	(Vertical OR Longitudinal) AND ((root OR tooth) NEAR/2 (fracture* OR injur*)) OR (craze NEAR/2 line*) OR (crazy NEAR/2 line*)
Intervention	‘Therapy’/Exp OR ‘Management’/Exp OR ‘Oral surgery’/Exp	Treatment* OR repair* OR management OR therap* OR bonding
